# Early-life iron deficiency persistently disrupts affective behaviour in mice

**DOI:** 10.1080/07853890.2023.2191003

**Published:** 2023-04-25

**Authors:** Anna Gundacker, Micaela Glat, Jonathan Wais, Peter Stoehrmann, Arnold Pollak, Daniela D. Pollak

**Affiliations:** a Department of Neurophysiology and Neuropharmacology, Center for Physiology and Pharmacology, Medical University of Vienna, Vienna, Austria; b Department of Neurosurgery, Medical University of Vienna, Vienna, Austria; c Department of Pediatrics and Adolescent Medicine, Medical University of Vienna, Vienna, Austria

**Keywords:** Iron deficiency, mouse behaviour, hippocampus, miRNA profile, depression, anxiety disorder

## Abstract

**Background/Objective:**

Iron deficiency (ID) is the most common nutrient deficiency, affecting two billion people worldwide, including about 30% of pregnant women. During gestation, the brain is particularly vulnerable to environmental insults, which can irrevocably impair critical developmental processes. Consequently, detrimental consequences of early-life ID for offspring brain structure and function have been described. Although early life ID has been associated with an increased long-term risk for several neuropsychiatric disorders, the effect on depressive disorders has remained unresolved.

**Materials and methods:**

A mouse model of moderate foetal and neonatal ID was established by keeping pregnant dams on an iron-deficient diet throughout gestation until postnatal day 10. The ensuing significant decrease of iron content in the offspring brain, as well as the impact on maternal behaviour and offspring vocalization was determined in the first postnatal week. The consequences of early-life ID for depression- and anxiety-like behaviour in adulthood were revealed employing dedicated behavioural assays. miRNA sequencing of hippocampal tissue of offspring revealed specific miRNAs signatures accompanying the behavioural deficits of foetal and neonatal ID in the adult brain.

**Results:**

Mothers receiving iron-deficient food during pregnancy and lactation exhibited significantly less licking and grooming behaviour, while active pup retrieval and pup ultrasonic vocalizations were unaltered. Adult offspring with a history of foetal and neonatal ID showed an increase in depression- and anxiety-like behaviour, paralleled by a deranged miRNA expression profile in the hippocampus, specifically levels of miR200a and miR200b.

**Conclusion:**

ID during the foetal and neonatal periods has life-long consequences for affective behaviour in mice and leaves a specific and persistent mark on the expression of miRNAs in the brain. Foetal and neonatal ID needs to be further considered as risk factor for the development of depression and anxiety disorders later in life.

**Key Messages**
1. Marginal reduction of gestational alimentary iron intake decreases brain iron content of the juvenile offspring.
2. Early-life ID is associated with increased depression- and anxiety-like behaviour in adulthood.
3. Reduction of maternal alimentary iron intake during pregnancy is reflected in an alteration of miRNA signatures in the adult offspring brain.

## Introduction

Iron deficiency (ID) is the most prevalent nutritional deficiency in the world [1,2]. Iron is considered an essential element for numerous biological processes including neurological development and function [3,4]. During pregnancy, the developing brain is particularly vulnerable to environmental insults, which can irrevocably impair critical developmental processes, such as cell proliferation, differentiation, synaptogenesis and myelination [5]. These data are of particular clinical relevance considering that ID is reported to affect about 30% of pregnancies worldwide, with significantly higher prevalence in lower-income regions [6]. While many of the dysfunctions resulting from chronic ID during adulthood are reversible by iron supplementation, ID during foetal life exerts persistent effects on brain structure and function [1,7–9].

Indeed, ID during the foetal and neonatal periods has been associated with an increased risk for a number of neuropsychiatric disorders later in life, prominently including autism and schizophrenia [10,11]. Several of the pathomechanisms underlying the dire consequences of foetal ID on the nervous system have been explored in experimental animal model systems in the last decades and revealed deranged monoamine neurotransmitter metabolism, deficits in neuronal and glial energy processing, impaired myelination and gene expression [12].

While alterations resulting from foetal ID been identified in several brain regions, a large body of evidence supports a specific vulnerability of the hippocampus, an area with high metabolic demands and expansive development in the perinatal period. With a prominent role in learning and memory [13–15], pathological alterations in the hippocampus have been robustly related to the cognitive deficits in children after early-life ID [12].

However, the hippocampus also plays a key role in the neural circuitry of depression and a correlation between ID and an increased risk of depressive disorder, including postpartum depression, has been proposed [5,6]. Pathomechanistically the relationship between ID and depression can be explained at several levels: (i) iron is essential for the proper function of a number of enzymes involved in neurotransmitter synthesis, including those related to the control of emotion and cognition, such as serotonin, norepinephrine and dopamine [16]; (ii) early-life ID induces short- and long-term abnormalities in hippocampal dendritic structure [17], monoaminergic and glutamatergic neurotransmission [18] and myelination [19] and (iii) impacts on the expression of brain-derived neurotrophic factor (BDNF) hereby disrupting hippocampal neurogenesis, intimately linked to depression and the response to antidepressant treatment [20]. Yet, the possible effects of early-life ID on the development of depression-like behaviour later in life, and its underlying mechanisms, have hitherto not been investigated.

Here, we designed a mouse model to test the hypothesis that marginal foetal and neonatal ID, resulting from a 50% reduction of maternal dietary availability during pregnancy and the early lactation period, is associated with a disruption of adult offspring depression- and anxiety-like behaviour. We comprehensively delineate the behavioural consequences of ID in our model, from the assessment of maternal behaviour and pup ultrasonic vocalizations in the early postpartum period, to depression- and anxiety-like behaviour in the adult offspring. The long-lasting consequences of early-life ID on the molecular composition of the brain are revealed by defining its miRNA signature in the adult hippocampus.

## Materials and methods

### Study protocol

**Figure 1.**
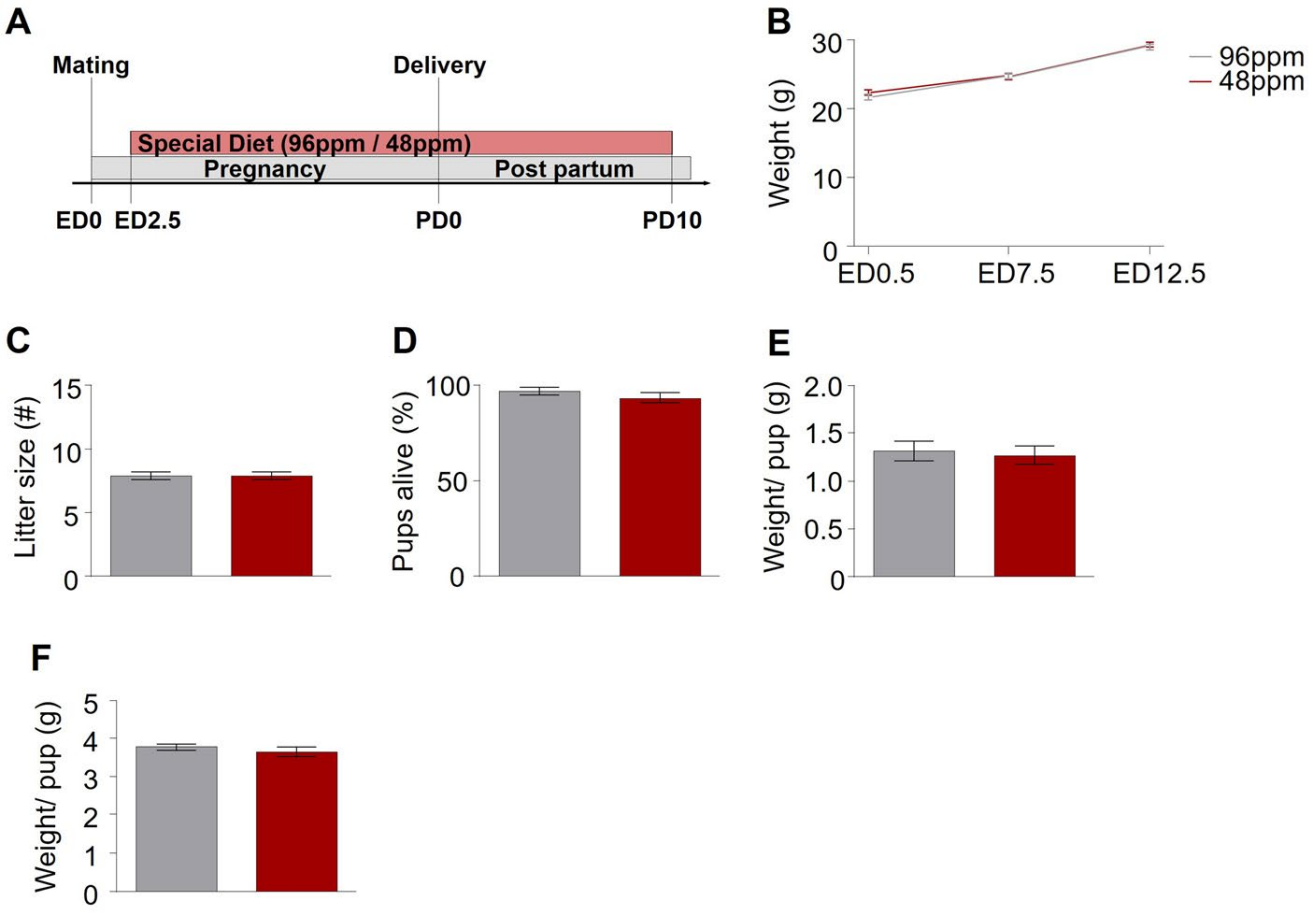
Paradigm for dietary iron restriction of pregnant mice. (A) Schematic depiction of the experimental timeline. No difference was detected in the (B) weight gain of mothers with an iron-deficient diet (IDD) (48 ppm) and iron-sufficient diet (ISD) (96 ppm) during pregnancy. (C) Litter size, (D) percentage of pups alive, and (E) average weight per pup did not differ between IDD and ISD treated animals on the day of delivery, as well as (F) average weight per pup on postnatal day 7 (P7) (*n*=38–43/group). All data are presented as mean ± S.E.M. Data were analysed using two-way ANOVA with repeated measures.

To address the long-lasting impact of early-life ID on offspring affective behaviour later in life, we employed a mouse model of dietary iron restriction in which dams received an iron-deficient diet (IDD) throughout pregnancy and during the early lactation phase (Figure 1(A)). Commercially available mouse breeding diets use a formulation containing 100 ppm (e.g. mouse breeding extrudate V1126-000 Ssniff®). An approximately 50% reduction of food iron concentration is considered adequate to maintain maternal weight gain and haemoglobin concentration in pregnant and lactating rodents [21,22]. Against this background, we decided to use a diet with 48 ppm as marginal IDD and 96 ppm as iron-sufficient diet (ISD) control, which would allow assessing the long-term consequences of gestational ID without unspecific biases of foetal growth retardation or maternal anaemia. From a translational perspective, this would correspond to a daily dietary intake of 13.5 mg of iron in pregnant women (50% reduction from the 27 mg/day) [23]. However, iron absorption may differ between rodents and people [24,25] and haeme versus non-haeme iron [26].

### Animals

All mice were kept under standard conditions in a temperature and humidity-controlled room with a 12 h light/dark cycle and food and water available *ad libitum*. The light intensity was set at 5–10 lux inside the cages. All animal experiments were carried out in accordance with the ARRIVE guidelines and the U.K. Animals (Scientific Procedures Act, 1986 and associated guidelines, EU Directive 2010/63/EU for animal experiments). Animal experiments described in this study were approved by the national ethical committee on animal care and use (2020-0.193.053; Bundesministerium für Wissenschaft und Forschung).

Two- to three-month-old male and female C57Bl/6N mice, purchased from Charles River (Sulzfeld, Germany) were used for timed mating as previously described [27]. The body weight of pregnant females was recorded on embryonic day (ED) 0.5, ED7.5 and ED12.5. On the day of delivery, postnatal day (PD) 0, the total number and the number of alive pups as well as the body weight of pups was assessed. Pups were weaned on PD21, separated by sex and maintained group-housed.

#### Experimental food

Alimentary iron restriction was achieved by supplying experimental mice with iron-deficient diet (48 ppm; Ssniff-Spezialdiäten GmbH, Soest, Germany) while control mice received iron-sufficient diet (96 ppm; Sniff) from ED2.5 until PD10 (Figure 1(A)).

### Behavioural analysis

Behavioural experiments were conducted during the light phase of the light/dark cycle. In each instance, animals were allowed to habituate to the experimental room for a minimum of 1 h prior to testing. Behavioural tests on adult offspring (2–4 months of age) were conducted in the order of increasing stress severity, observing an inter-test interval of at least 24 h [28]. Investigators were blinded to the experimental condition of the animals.

#### Maternal care behaviour

Maternal care behaviour was scored according to a previously published protocol [29]. Briefly, dams were recorded every day between PD1 and PD6 from 11 am-1 pm and from 3pm-5 pm using commercially available webcams (Logitech C525 HD Webcam, Microsoft LifeCam HD-3000, Trust Widescreen HD-Webcam, Creative LIVE! Cam Chat HD USB-Webcam). Behaviours were video-scored every 3 min and included the evaluation of pup licking/grooming, nest-building, nursing, and non-pup relevant parameters (eating/sleeping, self-grooming). Time spent displaying the individual behaviours was described as percentage (%) of total behavioural activity.

#### Ultrasonic vocalizations

On PD4 4 pups from each litter were randomly selected for the assessment of ultrasonic vocalizations (USVs) calls. To this end, pups were individually placed inside a noise-insulated box containing an USB Ultrasound microphone (Noldus Information Technologies, Wageningen, The Netherlands) for a period of 3 min. The obtained sonograms were analysed using the UltraVox XT software (Noldus) to calculate the number and amplitude of calls.

##### Sucrose preference test

The Sucrose Preference Test was adapted from a previously published protocol [30]. As such, three days before the test, mice were habituated to a 2% sucrose solution (Sigma-Aldrich, Vienna, Austria) for 48 h, followed by a period of 6 h of exposure to normal tap water. Mice were then food- and water-restricted for 18 h prior to the test session. The test consisted of presenting the animals with two identical bottles containing either 2% sucrose solution or tap water for a period of 3 h, after which the consumption rate of both liquids was determined. The relative sucrose preference (% of total liquid consumption) was calculated and used as indicator for hedonic behaviour [31].

#### Open field test

The open field test was conducted following the adaption of a protocol previously described [32]. Mice were placed in a transparent arena (27.3 cm × 27.3 cm) and allowed to freely explore the environment for 15 min. The movement of the animals was tracked using integrated laser beams inside the arena in combination with a analysis software (Activity Monitor v5, MedAssociates, Fairfax, VT, USA) and the total distance moved (cm) was used to assess their locomotor activity.

#### Light-dark box test

The light-dark box test was carried out as described [33] using a rectangular arena (27.3 cm× 27.3 cm) divided into two equal compartments. One compartment was strongly illuminated (250 lux), the other one was dim (maximum 5 lux). The location and movement of the animals was recorded using an automated system (Activity monitor, Med Associates) and the time spent in the light compartment (% of total time) was employed as indicator of anxiety-like behaviour.

##### Rotarod

The rotarod was performed following a published protocol [34]. Mice were tested for their performance of motor coordination using an automatic rotarod system for rodents (MedAssociates). They were placed onto a rotating drum (increasing in speed from 4 to 40 revolutions per minute over 6 min) for three consecutive trials, and the latency to fall off was measured by integrated laser beam detectors. Their average latency (s) to fall off the rotating drum was used to reflect motor coordination.

#### Forced swim test

The forced swim test was adapted from a published protocol [35]. In short, mice were placed inside a beaker filled with water (23 ± 1 °C) for 6 min and their movements were recorded employing an automated tracking software (VideoTrack v3, Viewpoint, Champagne au Mont d’Or, FR). The average immobility (% of total time), defined as the absence of movement in the final 4 min was calculated and used as proxy for behavioural despair indicating depression-like behaviour [36].

##### Tail suspension test

The tail suspension test was carried out as described [37]. Accordingly, mice were suspended by their tails using a commercial cubicle tail suspension system (MedAssociates). An automated monitoring system quantified the time spent immobile and mobile during the 6 min testing period. The duration of immobility (% of total time) was considered as indicator of behavioural despair, reflecting depression-like behaviour.

### Blood analysis

Mice were deeply anaesthetized with isoflurane and blood samples were collected through cardiac puncture of the right cardiac ventricle. Blood samples were kept at room temperature for at least 45 min before centrifugation at 1.200 *g* for 10 min for serum collection, or used immediately for haematology.

#### Haematology

Haematology was performed using an ADVIA 2120i system (Siemens, Erlangen, Germany). The system applies photometry, flow cytometry and impedance measurement to create standard hemograms.

#### Enzyme-linked immunosorbent assay (ELISA)

A commercially available mouse Soluble Transferrin Receptor (sTFR) ELISA Kit (MyBioSource, San Diego, CA, USA) was used. Samples were processed according to procedures recommended in the provider’s manual.

### Brain analyses

#### Prussian Blue staining

Animals were perfused with 20 mL phosphate buffer solution (PBS) 1x and 20 mL paraformaldehyde (PFA) solution 4%. Brains were extracted and fixed in PFA 4% solution overnight at 4 °C and kept in 30% sucrose solution for 3 days at 4 °C. After embedding in Optimal Cutting Temperature (O.C.T.) (Tissue Plus, O.C.T. Compound, Scigen Scientific Gardena, CA, USA) medium they were frozen in liquid nitrogen. Perfused brains were cut on a cryostat (Leica®, Germany) in 40 µm slices and incubated with a mixture of equal parts of 4% potassium ferrocyanide and 4% hydrochloric acid (Polyscience Inc.® Prussian Blue Staining Kit), followed by staining with 3,3′-Diaminobenzidine (DAB) (Vector® DAB Peroxidase Substrate Kit). Images were taken on a Nikon® (Nikon, Tokyo, Japan) Eclipse E600 Fluorescence Microscope with a 10x objective and analyzed (density) using ImageJ (U. S. National Institutes of Health, Bethesda, MD, USA, https://imagej.nih.gov/ij/).

#### NeuN staining

Animals were perfused, brains frozen and

sectioned in 30 µm coronal slices on a cryostat (CM1950, Leica, Wetzlar, Germany) and stored at −20 °C in cryoprotectant solution (30% glycerol, 30% ethylene glycol, and 40% PBS 1x) until further use. Slices were stained with a mouse monoclonal antibody against the neuronal nuclear antigen NeuN (Chemicon®, MAB377, MS X NeuN, 1:1000) and a secondary polyclonal goat anti mouse 488 antibody (Thermo Fisher Scientific®, Alexa Fluor® 488 goat anti-mouse IgG (H + L), A11029, 1:1000). Images were taken on Zeiss® (Oberkochen, Germany) Axiovert 200 M Fluorescence/Live cell Imaging Microscope with a 10x and 20x objective and analysed (cells/mm^2^) using ImageJ.

#### Neurogenesis analysis

Progenitor cell proliferation was analysed based upon published BrdU ((+)-5′ Bromo-2′-deoxyuridine) injection protocols [38]. Mice were administrated 10 µl/g body weight of 50 mg/kg BrdU (Sigma-Aldrich, St. Louis, MO, USA) dissolved in 0.9% NaCl (i.p.) four times in 2 h intervals and perfused 24 h after the last injection. Coronal brain sections (30 µm) containing the ­hippocampus were generated and every 10^th^ section of the entire rostro-caudal span was used for immunofluorescence-histochemistry. To this, brain slices were stained with a mouse anti-BrdU antibody (Bio-Rad AbD Serotec, United Kingdom; 1:300) and a 488 goat anti-mouse (Thermofisher Scientific, Waltham, MA, USA, 1:500). Images were acquired on a Zeiss® (Oberkochen, Germany) Axiovert 200 M Fluorescence/Live cell Imaging Microscope with a 10x and 20x objective and analysed using ImageJ.

#### miRNA sequencing

Animals were killed by cervical dislocation and hippocampal samples were collected, rapidly frozen in liquid nitrogen and stored at −80 °C, until further processing. miRNA was extracted using the miRNAeasy mini kit (74104, Qiagen, Hilden, Germany) following the manufacturer’s instructions. Concentration and purity of RNA were determined using a Nanodrop photometer (NanoPhotometer TM 7122 V2.3.1, IMPLEN, Munich, Germany).

miRNA-seq libraries were prepared with the TruSeq Stranded miRNA LT sample preparation kit (Illumina, San Diego, CA, USA) using Sciclone and Zephyr liquid handling workstations (PerkinElmer, Waltham, MA, USA). Expression profiling libraries were sequenced on a HiSeq 4000 instrument (Illumina, San Diego, CA, USA). Transcriptome analysis was performed with the Tuxedo suite. For each sample, NGS reads passing vendor quality filtering were aligned to the UCSC Genome Browser mm10 flavour of the Genome Reference Consortium GRCm38 assembly with TopHat2 (v2.1.1) [12]. Cufflinks (v2.2.1) allowed for transcriptome assembly, on the basis of spliced read alignments and the reference transcriptome, as well as raw transcript quantification [13]. Cuffdiff (included in Cufflinks v2.2.1) was used for differential expression calling [14]. miRNAs with a false discovery rate FDR ≤ 0.05 and a log2-fold change ≥ + 1.5 or ≤ − 1.5 for up- and downregulation, respectively, were considered as significantly differentially expressed.

Target gene search for significantly differentially expressed miRNAs 200a and 200b was performed with TargetScanMouse (Version 8.0). Gene ontology enrichment analysis for common target genes of miRNA 200a and 200b was carried out for biological processes using the freely available software tool Enrichr [39,40].

### Statistical analysis

All analyses were performed by an investigator blinded to the experimental condition of the animals. N numbers, full statistics and Ρ values are reported for each main effect and all interactions are listed where relevant. Sample sizes were determined according to own experience and data provided in literature [27,28,37]. G-power analysis was conducted based upon a two-tailed computation for sample size prediction with a confidence level α = 0.05 and power = 0.8. For maternal care experiments the analysis provided non-centrality parameter δ (3.8969155), critical t (2.5705818) and a required sample size of 6 per group resulting in an actual power of 0.872. For offspring behavioural experiments non-centrality parameter δ (2.9973947), critical t (2.0738731) and a required sample size of 23 per group was calculated resulting in an actual power of 0.817. All statistical analyses were performed using GraphPad Prism 7 (La Jolla, CA, USA). Data were tested for normality using the Kolmogorov–Smirnov test prior to further statistical evaluation. Outliers were removed by using the Tukey’s boxplot method. Student’s *t*-test was applied when two groups with normally distributed data were compared. For comparisons between groups in a repeated measures design, two-way ANOVA with repeated measures was applied.

For all analyses, *p* < 0.05 was considered statistically significant.

The volcano plot was prepared using Python (3.11.1; Python Software Foundation).

## Results

### Marginal foetal/neonatal ID does not alter pre- or postnatal growth but reduces brain iron content in juvenile mice

We first confirmed that mothers receiving IDD (48 ppm FeSO_4_; *n*=38) and ISD (96 ppm FeSO_4_; *n*=43) did not differ in weight gain during pregnancy (Figure 1(B)), excluding a general impact of IDD on overall metabolism and physiology of the dams during gestation. Furthermore, no effect of IDD on the number of pups per litter (Figure 1(C)), the percentage of live pups per litter (Figure 1(D)), or their weight at birth (Figure 1(E)) was observed, corroborating that IDD did not lead to intrauterine growth retardation/death or foetal demise. To prevent a bias in thriving due to the considerably rising iron demands during the period of substantial postnatal growth, all animals were switched to ISD on PD10.

**Figure 2.**
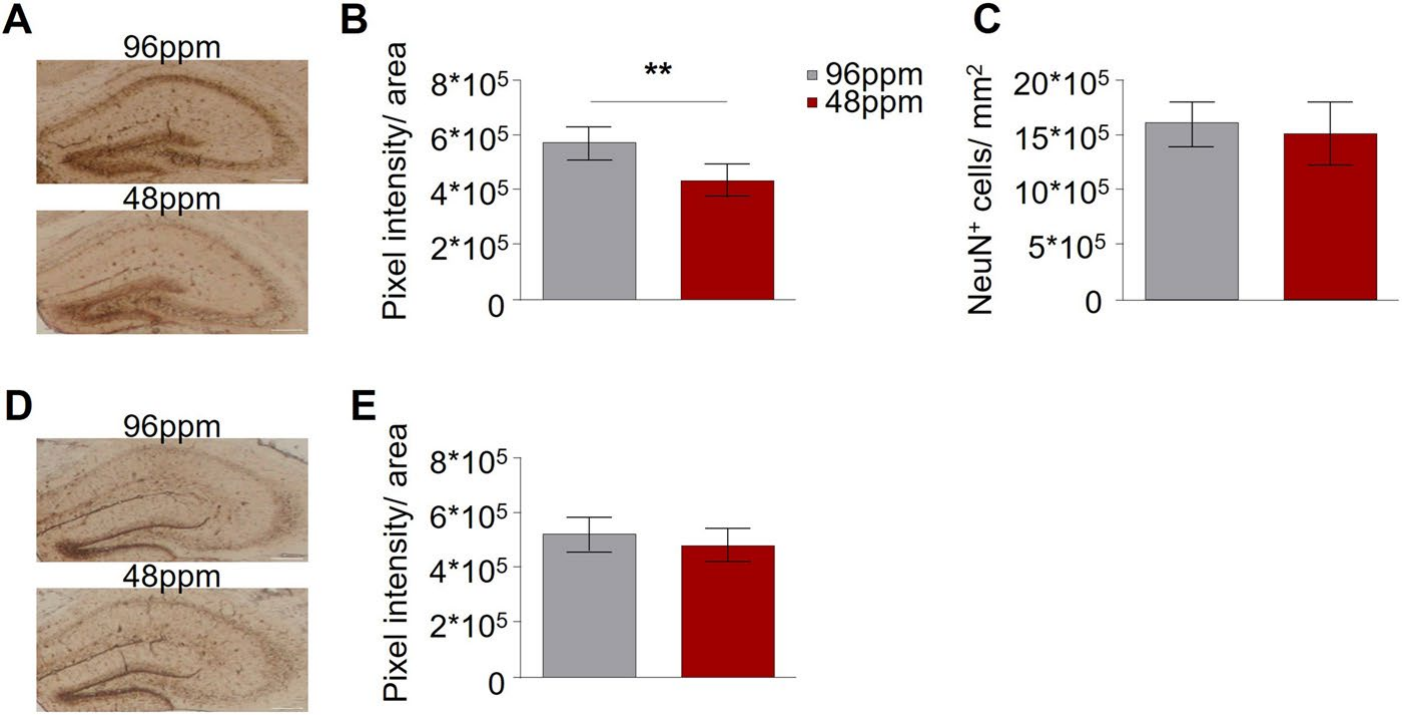
Maternal alimentary iron restriction during pregnancy and lactation reduces the iron content in the pup brain. (A) Representative images of Prussian Blue staining of hippocampal sections of postnatal day 11 (PD11) offspring from mothers provided with iron-deficient diet (IDD) (48 ppm) or iron-sufficient diet (ISD) (96 ppm) during pregnancy and lactation. Scale bars: 200 µm, 10x magnification. (B) Quantification of iron levels in the hippocampus revealed a significant difference between IDD and ISD PD11 offspring (*n*=6/group). (C) Comparison of NeuN-positive cells per mm^2^ indicated no difference in the amount of neuronal cells in the hippocampus of ISD and IDD offspring on PD11 (*n*=6/group). (D) Representative images of hippocampal sections with Prussian Blue staining in adult IDD and ISD offspring. Scale bars: 200 µm, 10x magnification. (E) Quantification showed no difference in hippocampal iron levels between IDD and ISD adult offspring (*n*=5/group). All data are presented as mean ± S.E.M. Data were analysed using two-way ANOVA with repeated measures; **p < 0.01.

In order to verify that our protocol of marginal ID indeed reduced offspring brain iron content, pups were sacrificed on PD11 and non-haeme iron was determined in brain (hippocampal) sections of IDD and ISD offspring (*n*=6). Prussian blue staining revealed a significant reduction of iron content in the hippocampus of IDD pups (Figure 2(A,B)). Immunohistochemistry using the neuronal marker NeuN showed that the neuronal density was comparable between IDD and ISD offspring (Figure 2(C)), demonstrating that marginal foetal ID did not lead to neuronal loss or hamper neurogenesis.

We next asked whether the direct effect of IDD on brain iron content was reversible, since IDD animals were reared on a diet with adequate iron content from P10 onwards. No differences in hippocampal iron content between adult IDD and ISD animals was detected (Figure 2(D,E)), confirming that replenishment of iron stores in the juvenile period is sufficient for brain iron content to reach control levels in adulthood. Paralleling these observations, is the documentation of normal levels of serum sTFR and red and white blood cell count in adult IDD offspring (Supplementary Table 1).

### Gestational ID impairs spontaneous maternal care behaviour postpartum

**Figure 3.**
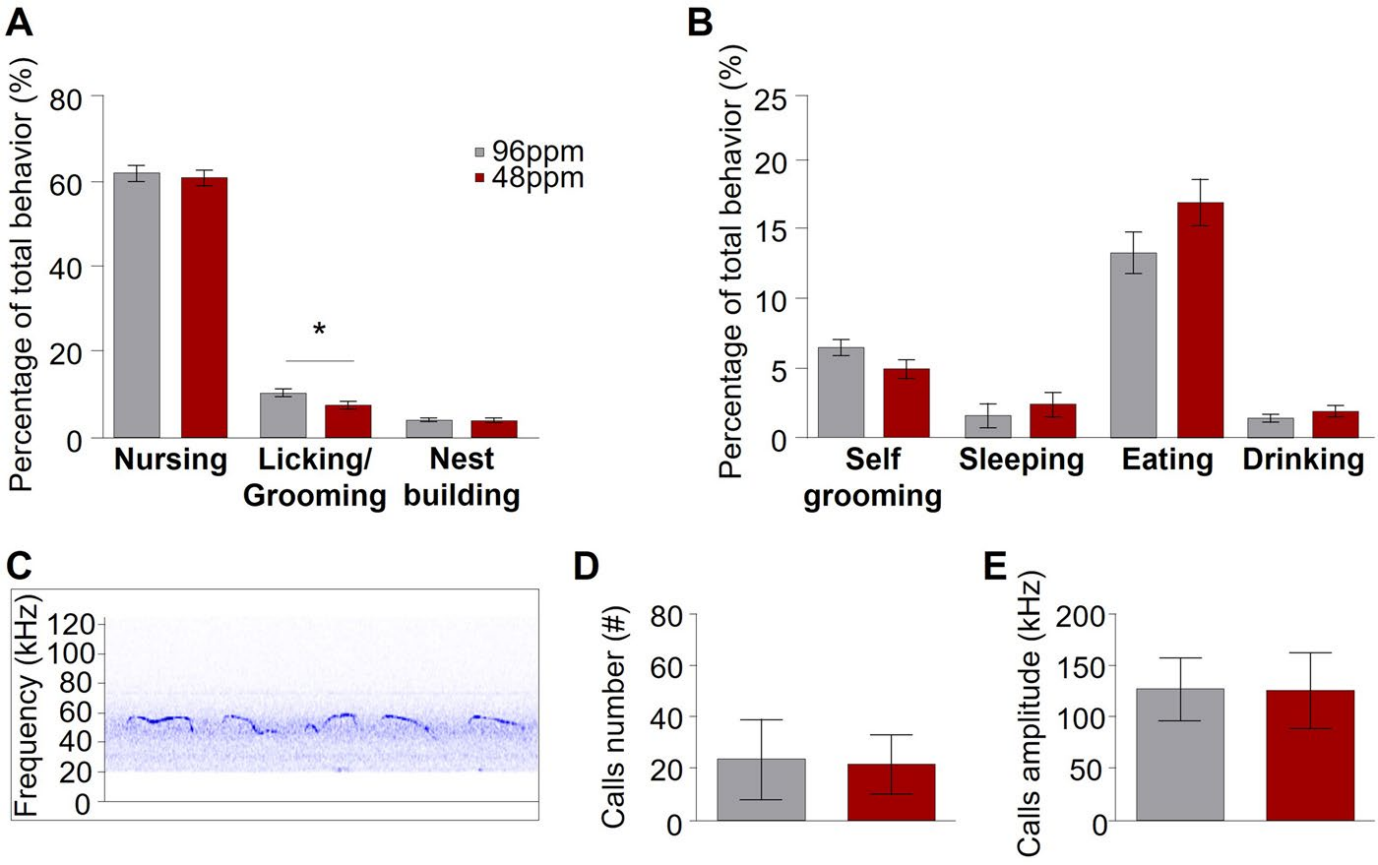
Reduced postnatal spontaneous maternal care behavior upon dietary ID during pregnancy in mice. Percentage of different (A) pup-directed (nursing, licking and grooming, and nest building) and (B) non pup-directed (self-grooming, sleeping, eating, and drinking) behaviours from postnatal day 1 (PD1) to PD6 of mothers provided with iron-deficient diet (IDD) (48 ppm) or iron-sufficient diet (ISD) (96 ppm) (*n*=8/group). IDD mothers showed significantly reduced licking and grooming behaviour but no other differences between groups were observed. (C) Exemplary depiction of a trace of recorded ultrasonic vocalizations (USVs) recorded from pups on PD4. (D) Mean number and (E) amplitude of USV calls of IDD and ISD pups on PD4 revealed no difference between groups (*n*=19/group). All data are presented as mean ± S.E.M. Data were analysed using mixed model ANOVA with repeated measures and Student’s *t*-test where appropriate; *p < 0.05.

Although several studies have reported neuropsychiatric phenotypes in offspring after early-life ID in animal models [10,11,18], the consequences of ID during pregnancy and lactation on the care the dam provides for her pups, has remained unexplored. We here addressed this question and evaluated spontaneous maternal care behaviour in IDD and ISD mothers by home cage observation during the first six postnatal days (*n*=6). We found a selective and specific reduction in the relative time IDD mothers spent licking and grooming their pups but no other pup-directed (nursing, and nest building) or non-pup-directed (self-grooming, sleeping, eating, and drinking) behavioural displays (Figure 3(A,B)).

Maternal care behaviour is a form of social behaviour based upon the interaction between a mother and her offspring. Communication from the pups to their mother is mediated through pup ultrasonic vocalizations. However, we found no alterations of ultrasonic vocalizations in IDD pups as both the number of emitted ultrasonic calls and their amplitude was comparable between IDD and ISD pups (Figure 3(C–E)), indicating that a communicatory deficit from the side of the pups is unlikely at this developmental stage and that IDD pups are able to efficiently interact with their mother.

### Early-life ID persistently disrupts affective behaviour

**Figure 4.**
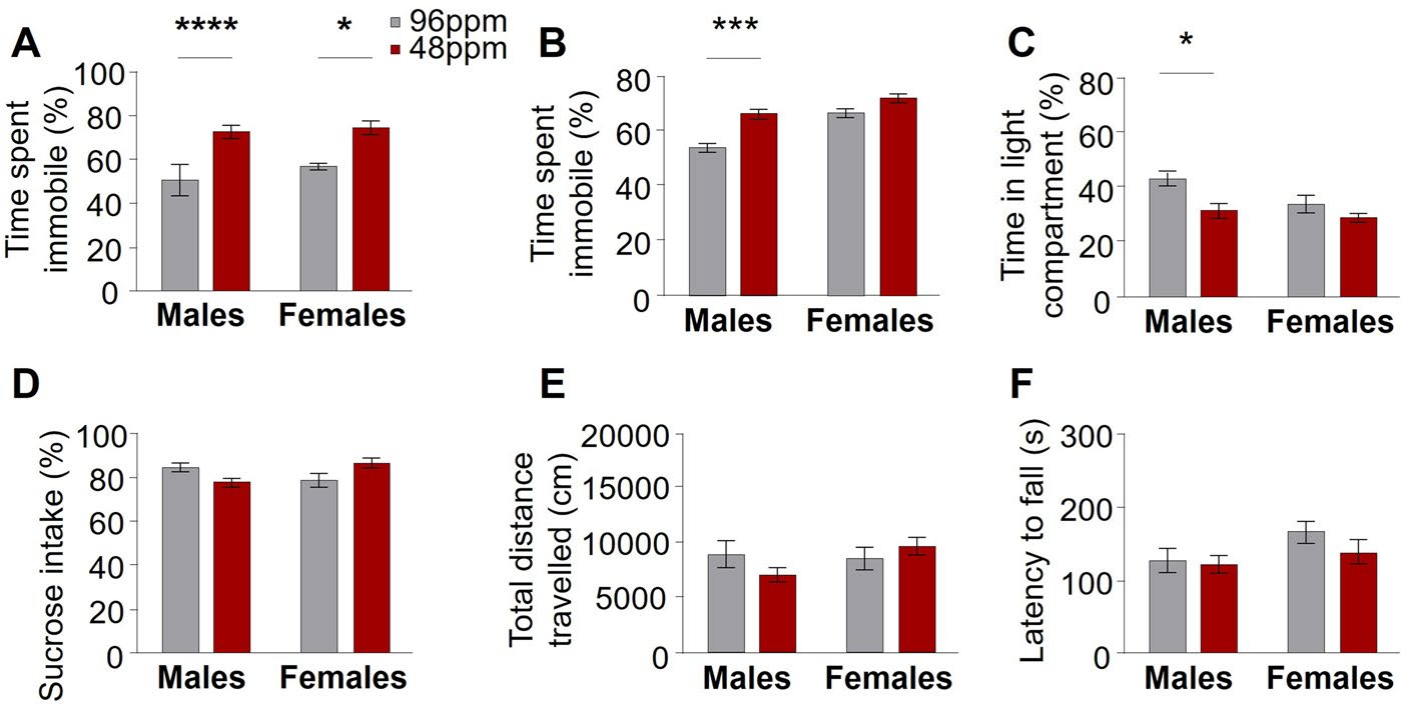
Increased depression-like behaviour in adult mice after early-life ID. Significant differences in behavioural despair in (A) the forced swim test in male and female and (B) the tail suspension test in male offspring from mothers provided with iron-deficient diet (IDD) (48 ppm) compared to iron-sufficient diet (ISD) (96 ppm) during pregnancy and lactation. (C) A significant decrease of time spent in the light compartment of the light/dark box in IDD male mice demonstrates increased anxiety-like behaviour. No differences between groups was found in anhedonic behaviour in the (D) sucrose preference test (percentage of sucrose preference), (E) exploratory activity in the open field test (total distance travelled) and (F) motor coordination in the rotarod (latency to fall off) (*n*=20–28/group). All data are presented as mean ± S.E.M. Data were analysed using two-way ANOVA; *p < 0.05, **p < 0.005, ***p < 0.001, ****p < 0.0001.

Well-established and widely accepted behavioural tests for the robust assessment of depression and anxiety like behaviour in rodents exist [41]. Here, we tested adult female and male animals (P90-P120; *n*=20–28) with a history of foetal/neonatal ID and control animals in a standard battery of highly specific behavioural paradigms: the sucrose preference test, which measures anhedonic behaviour by assessing the preference for consumption of a sweet solution over water; the forced swim test and the tail suspension test, both evaluating behavioural despair, in an inescapable aversive situation; the light-dark box, which gauges anxiety-like behaviour by quantifying the time an animal spends in an anxiogenic, brightly illuminated compartment versus time spent in a less fear-inducing, dark and closed compartment. Throughout all tests, we consistently found an increase of depression- and anxiety-like behaviour in animals after foetal/neonatal ID. This was reflected in significantly increased behavioural despair (immobility) in the forced swim and tail suspension tests (Figure 4(A,B)), and augmented anxiety-like behaviour in the light-dark box (reduction in time spent in the light compartment; Figure 4(C)) compared to control mice. There was no significant difference in the preference for sucrose in the sucrose preference test (Figure 4(D)). Importantly, no alterations in the general exploratory behaviour in the open field test and locomotor activity in the rotarod in adult IDD offspring (Figure 4(E,F)) were found, ruling out an unspecific performance bias and corroborating the relevance of the results in the tests specifically assessing affective behaviours.

### Adult hippocampal progenitor cell proliferation remains intact after foetal and neonatal ID

**Figure 5.**
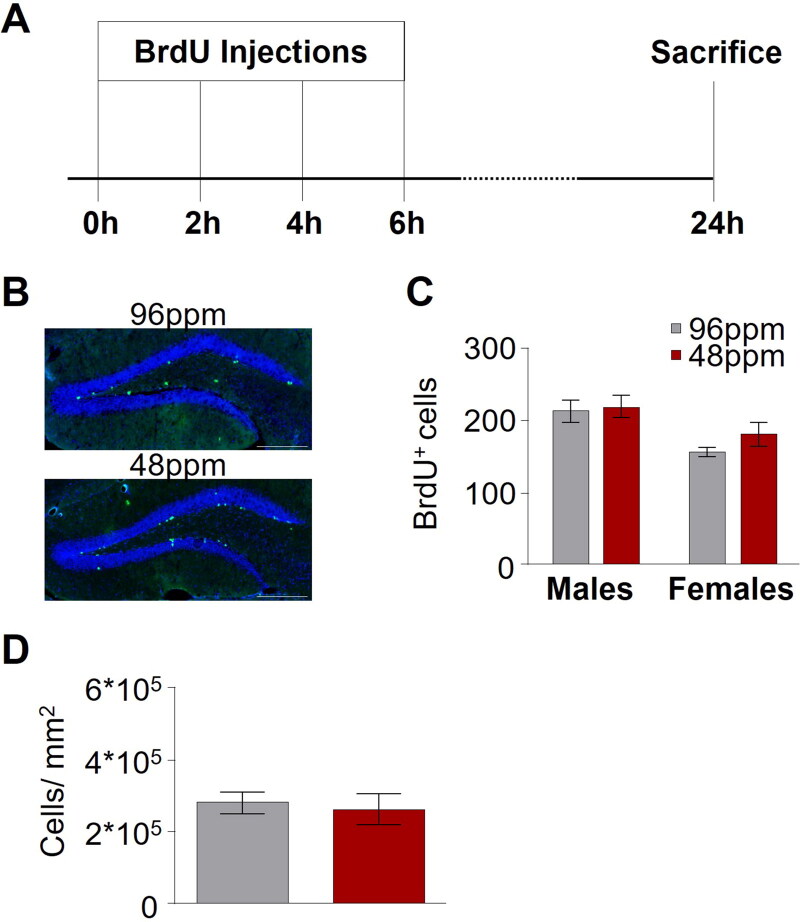
Adult hippocampal neurogenesis is not altered upon after early-life ID. (A) Schematic depiction of the protocol timeline for the administration of BrdU ((+)-5’ Bromo-2’-deoxyuridine). (B) Representative images of BrdU staining in offspring of mothers provided with an iron-deficient diet (IDD) (48 ppm) or iron-sufficient diet (ISD) (96 ppm) during pregnancy and lactation (until postnatal day 10). Scale bars: 200 µm, 20x magnification. (C) Quantification of BrdU^+^ cells in the hippocampus (*n*=5–9/group), as well as (D) comparison of NeuN-positive cells per mm^2^ in hippocampal areas indicated no difference between ISD and IDD animals (*n*=8/group). All data are presented as mean ± S.E.M. Data were analysed using two-way ANOVA and Student’s *t*-test where appropriate.

There is a strong body of evidence, across various experimental model systems and also in human studies supporting a role for adult hippocampal neurogenesis in depression and in the response to antidepressant treatment [42–44]. We thus set out to test whether a derangement of hippocampal neurogenesis could be associated with the behavioural deficits in mice after foetal/neonatal ID using 5-Bromo-2 Deoxyuridine (BrdU) labelling of newly generated cells in the dentate gyrus to assess the proliferation of hippocampal progenitor cells (Figure 5(A)). We found no difference in the number of newly born cells between IDD and ISD animals (Figure 5(B,C)), and further confirmed that also the total number of neurons, as revealed by NeuN immunohistochemistry, was comparable between groups (Figure 5(D)).

### A distinct miRNA expression signature reflects the long-lasting consequences of early-life ID in the brain

The early environment can deflect brain development with persistent consequences, including an increased risk for the development of neuropsychiatric disorders, by inducing structural alterations and/or by deregulating gene networks. miRNAs are critical regulators of gene expression [45] and involved in the pathophysiology of several psychiatric disorders, such as depression [46,47]. Importantly, iron is pivotally engaged in the activity of the miRNA pathway [48]. Against this background we decided to conduct an unbiased miRNA sequencing (miRNAseq) of hippocampal tissue of adult animals after foetal ID for an unbiased screening of the molecular correlates accompanying the behavioural deficits.

**Figure 6.**
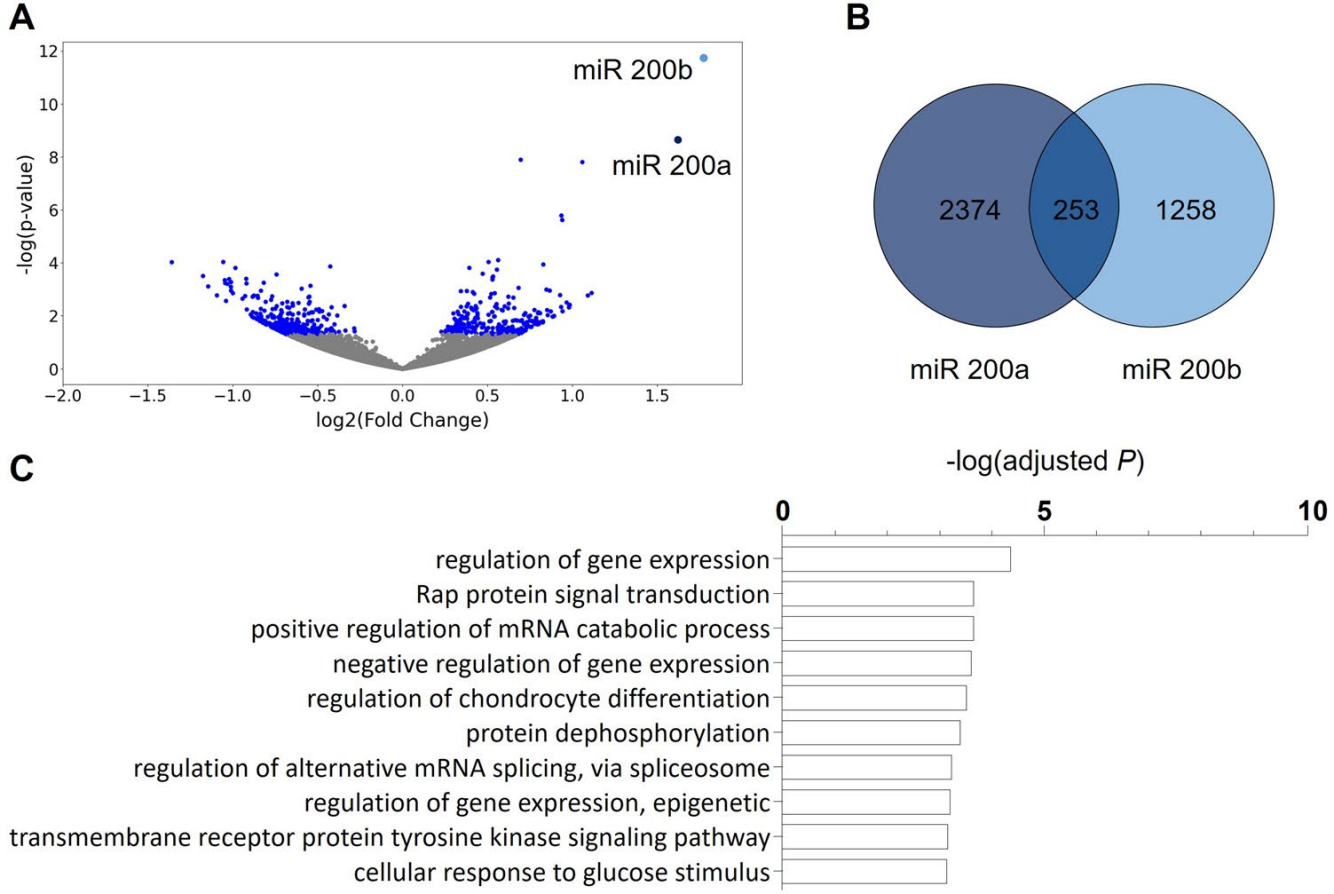
The miRNA expression pattern in the adult hippocampus is modulated by early-life ID. (A) Results of miRNA sequencing in hippocampal tissue of offspring of mothers with an iron-deficient diet (IDD) (48 ppm) and iron-sufficient diet (ISD) (96 ppm) revealed 530 differentially expressed miRNAs (blue dots: *q* < 0.05). Only two miRNAs, miR 200a and miR 200b, had a log2-fold changes of ≥ + 1.5 or ≤ − 1.5. (B) TargetScanMouse (Version 8.0) revealed 2627 target genes of miR 200a and 1511 target genes of miR 200b. Of these, 253 genes were targeted by both miR 200a and miR 200b, depicted by a Venn diagram (Venny 2.1.0). (C) Results of an enrichment analysis of genes targeted by both miR 200a and miR 200b revealed the involvement in a variety of different biological processes (white bars; Enrichr, GO: Biological processes).

After statistical correction for multiple testing we identified 530 miRNAs that were significantly differentially (false discovery rate FDR ≤ 0.05) expressed between IDD and ISD adult offspring. Of those 284 miRNAs were upregulated and 246 miRNAs were downregulated in hippocampal tissue of animals with a history of early life ID in comparison to controls (Figure 6(A)).

Considering only those miRNAs with log2-fold changes ≥ + 1.5 or ≤ − 1.5 reveals a selective and specific effect of early life ID on two miRNAs of the same family, miR 200a and miR 200b, both located on the mouse chromosome 4. Target gene prediction revealed 253 shared target genes of miRNA 200a and miRNA 200b (Figure 6(B); Supplementary Table 2). Further bioinformatic analysis of the biological function (GO terms, Figure 6(C)) of the predicted shared targets showed a distinct number of significantly enriched gene sets. Intriguingly, ‘regulation of gene expression’ was revealed as the most significantly enriched function, alongside other gene sets relating to the control of gene expression.

## Discussion

Early-life ID has been previously implicated in alterations of neurocognitive development and identified as long-term risk factor for some neuropsychiatric disorders [6,14,18]. Depression is a highly prevalent psychiatric disorder affecting an estimated 10–15% of the population worldwide, which constitutes a major challenge in global health management with significant socioeconomic repercussions. However, no information on the role of foetal/neonatal ID on depression and anxiety in adulthood is available to date and the role of maternal behaviour as a possible ‘second hit’ contributing to the phenotype resulting from early-life ID has remained unexplored.

In this study we showed in a mouse model that even marginally reduced availability of alimentary iron during pregnancy and lactation is sufficient to bias foetal brain development towards an adverse outcome for mental health, specifically depression, later in life. Although a major part of brain development occurs towards the end of pregnancy, the present study design restricted dietary iron intake throughout the entire gestational period, in order to more closely mimic the situation in the human population where the availability of iron in the diet is unlikely to change during the course of pregnancy. The presented preclinical data shows that only 50% reduction of dietary iron intake during pregnancy reduces iron content of the juvenile offspring brain and leads to long-lasting implications for affective behaviour in adult offspring, defined by a phenotypic profile of increased depression and anxiety-like behaviour, but without persisting effects on iron stores and serum iron indices, or haematological consequences. However, especially the differences in iron absorption and bioavailability between rodents and people have to be considered as caveat for the translation of the presented findings.

Importantly, we also found that gestational ID impaired spontaneous maternal care behaviour as IDD dams showed lower levels of licking/grooming behaviour than controls. Maternal care behaviour critically shapes offspring development with long-lasting consequences for brain function and behavioural outcomes, most prominently emotional behaviour and stress susceptibility, later in life [49–51]. Specifically, maternal licking/grooming, the major source of tactile stimulation of the newborn rodent, modulates the development of endocrine, emotional and cognitive responses to stress [52,53], herby importantly biasing the risk for the development of affective disorders, including depression and anxiety disorders [49,54,55]. Hence, it cannot be excluded that the behavioural phenotype of IDD offspring is, at least in part, a result of the reduced care behaviour received in the early postnatal period. Considering that USVs were unaltered in IDD pups it is likely that the deficient care behaviour of the mothers originates from a direct impact of dietary iron restriction during pregnancy on the maternal brain, rather than inadequate motivation by pup stimuli. However, the pathways involved and the corresponding impact on the brain of IDD dams remains to be explored in future studies. From a translational perspective, the reduction of care behaviour in IDD mothers can be considered within the known association between ID/ ID anaemia during pregnancy and an elevated risk of postpartum depression [56–60], a psychiatric condition, also reflected in the inability of the mother to appropriately care for her baby.

The investigation of the neural correlates of augmented depression-like behaviour in the adult IDD offspring brain revealed that foetal/neonatal ID is not altering cellular processes of neurogenesis or neuronal differentiation in adult life, which had been previously associated with depression-like behaviour in several animal models [61–63]. However, results of the present study reveal a specific and selective dysregulation of two miRNAs, miR 200a and miR 200b in hippocampal tissue of adult IDD offspring. This observation is relevant considering the large body of evidence supporting a significant role of miRNAs in brain development [64] and emerging literature on the function of miRNAs in the control of behaviour and emotion, particularly in psychiatric disorders in which this regulation is impaired [65]. Whereas several individual miRNAs and miRNA families have been associated with depression and/or antidepressant effects, including miR 16 [66], miR 155 [67], miR 182 [68], miR 132 [69], a role for the miR 200 family has not yet been described in this context. As such, a defined involvement for miR 200a and miR 200b in depression-like behaviour remains to be explored. However, several of the target genes, some of which by themselves act as regulators of gene expression (such as the transcription factors *jun* and *atf3*) have been demonstrated to be relevant to psychiatric disorders (including *nr3c1*, the glucocorticoid receptor [70,71]; *ntrk2,* the receptor for brain-derived neurotrophic factor; [72,73] and *cnr1*, the cannabinoid receptor 1 [74,75]). Hence, miR 200a and miR 200b and their target genes may constitute a gene regulatory network that forms the molecular basis for the pathophysiological events underlying the consequences early-life ID on affective behaviour.

The current study opens up several new fronts, not only for translational and clinical research questions, but also for further preclinical experiments. As such, the molecular and functional consequences of deranged miRNA 200a and 200b expression should be investigated, including direct effects on the expression of the respective target genes. Cross-fostering study designs could be employed to clearly demark the impact of maternal care behaviour, respectively its deficiency, in our model and discriminate it from the direct consequences of iron restriction on the foetal brain *in utero*. Moreover, an impact of foetal/neonatal ID on other brain regions forming part of the neural circuit of depression, including the prefrontal cortex and the amygdala, should be examined in animal models

Collectively, the present observations suggest that monitoring maternal alimentary iron intake should extend beyond controlling clinical and laboratory parameters of anaemia to ensure optimal foetal brain development and reduce the overall prevalence of depressive disorders. Future clinical studies should be specifically targeted to test the translatability of the present findings and explore a correlation between foetal ID and affective disorders. If confirmed in a clinical setting, this would propose a new dimension to be considered for public antenatal care programs, to tightly survey and ensure adequate nutritional supply of iron for pregnant women. Under these conditions, children with a confirmed history of iron restriction in early life should be considered to carry a higher risk for the development of affective disorders.

## Conclusion

Overall, our data provide the first preclinical study showing that marginal restriction of iron availability during foetal life sets the path for the later development of affective disorders and permanently alters the molecular composition. These observations set the foundations for future clinical studies to specifically test the role of foetal ID as risk factor for the development of depression and anxiety disorders later in life.

## Supplementary Material

Supplemental Material

Supplemental Material

## Data Availability

The data reported in the findings of this study are available from the corresponding author upon request.

## References

[1] Lozoff B, Beard J, Connor J, et al. Long-lasting neural and behavioral effects of iron deficiency in infancy. Nutr Rev. 2006;64(5 Pt 2): s34–43. discussion S72-91. Available: http://www.pubmedcentral.nih.gov/articlerender.fcgi?artid=1540447&tool=pmcentrez&rendertype=abstract 16770951 10.1301/nr.2006.may.S34-S43 PMC1540447

[2] Youdim MBH. Brain iron deficiency and excess; cognitive impairment and neurodegeneration with involvement of striatum and hippocampus. Neurotox Res. 2008;14(1):45–56. http://www.ncbi.nlm.nih.gov/pubmed/18790724 18790724 10.1007/BF03033574

[3] Beard JL, Connor JR, Jones BC. Iron in the brain. Nutr Rev. 1993;51(6):157–170. 8371846 10.1111/j.1753-4887.1993.tb03096.x

[4] Lozoff B, Brittenham GM, Wolf AW, et al. Iron deficiency anemia and iron therapy effects on infant developmental test performance. Pediatrics. 1987;79:981–995. http://www.ncbi.nlm.nih.gov/pubmed/2438638 2438638

[5] Rice D, Barone S. Critical periods of vulnerability for the developing nervous system: evidence from humans and animal models. Environ Health Perspect. 2000;108(Suppl):511–533. http://www.pubmedcentral.nih.gov/articlerender.fcgi?artid=1637807&tool=pmcentrez&rendertype=abstract 10852851 10.1289/ehp.00108s3511 PMC1637807

[6] Black MM, Quigg AM, Hurley KM, et al. Iron deficiency and iron-deficiency anemia in the first two years of life: strategies to prevent loss of developmental potential. Nutr Rev. 2011;69 Suppl 1:S64–S70. 22043885 10.1111/j.1753-4887.2011.00435.x

[7] Felt BT, Lozoff B. Brain iron and behavior of rats are not normalized by treatment of iron deficiency anemia during early development. J Nutr. 1996;126(3):693–701. 8598555 10.1093/jn/126.3.693

[8] Bastian TW, Von Hohenberg WC, Mickelson DJ, et al. Iron deficiency impairs developing hippocampal neuron gene expression, energy metabolism, and dendrite complexity. Dev Neurosci. 2016;38(4):264–276. 27669335 10.1159/000448514 PMC5157120

[9] Greminger AR, Lee DL, Shrager P, et al. Gestational iron deficiency differentially alters the structure and function of white and gray matter brain regions of developing rats. J Nutr. 2014;144(7):1058–1066. 24744313 10.3945/jn.113.187732 PMC4056646

[10] Insel BJ, Schaefer CA, McKeague IW, et al. Maternal iron deficiency and the risk of schizophrenia in offspring. Arch Gen Psychiatry. 2008;65(10):1136–1144. 18838630 10.1001/archpsyc.65.10.1136 PMC3656467

[11] Schmidt RJ, Tancredi DJ, Krakowiak P, et al. Maternal intake of supplemental iron and risk of autism spectrum disorder. Am J Epidemiol. 2014;180(9):890–900. 25249546 10.1093/aje/kwu208 PMC4207718

[12] Georgieff MK. Iron deficiency in pregnancy. Am J Obstet Gynecol. 2020;223(4):516–524. 32184147 10.1016/j.ajog.2020.03.006 PMC7492370

[13] Fretham SJB, Carlson ES, Georgieff MK. The role of iron in learning and memory. Adv Nutr. 2011;2(2):112–121. 22332040 10.3945/an.110.000190 PMC3065765

[14] Radlowski EC, Johnson RW. Perinatal iron deficiency and neurocognitive development. Front Hum Neurosci. 2013;7:1–11. 24065908 10.3389/fnhum.2013.00585 PMC3779843

[15] Geng F, Mai X, Zhan J, et al. Impact of fetal-neonatal iron deficiency on recognition memory at two months of age. J Pediatr. 2015;167(6):1226–1232. 26382625 10.1016/j.jpeds.2015.08.035 PMC4662910

[16] Burhans MS, Dailey C, Beard Z, et al. Iron deficiency: differential effects on monoamine transporters. Nutr Neurosci. 2005;8(1):31–38. 15909765 10.1080/10284150500047070

[17] Pisansky MT, Wickham RJ, Su J, et al. Iron deficiency with or without anemia impairs prepulse inhibition of the startle reflex. Hippocampus. 2013;23(10):952–962. 23733517 10.1002/hipo.22151 PMC3888485

[18] Unger EL, Hurst AR, Georgieff MK, et al. Behavior and monoamine deficits in prenatal and perinatal iron deficiency are not corrected by early postnatal moderate-iron or high-iron diets in rats. J Nutr. 2012;142(11):2040–2049. 22990465 10.3945/jn.112.162198 PMC3498975

[19] Connor JR, Menzies SL. Relationship of iron to oligodendrocytes and myelination. Glia. 1996;17:83–93. 8776576 10.1002/(SICI)1098-1136(199606)17:2<83::AID-GLIA1>3.0.CO;2-7

[20] Tran PV, Fretham SJB, Carlson ES, et al. Long-Term reduction of hippocampal Brain-Derived neurotrophic factor activity after Fetal-Neonatal iron deficiency in adult rats. Pediatr Res. 2009;65(5):493–498. 19190544 10.1203/PDR.0b013e31819d90a1 PMC2715440

[21] Kochanowski BA, Sherman AR. Iron status of suckling rats as influenced by maternal diet during gestation and lactation. Br J Nutr. 1983;49(1):51–57. 6821689 10.1079/bjn19830010

[22] Koenig MD, Tussing-Humphreys L, Day J, et al. Hepcidin and iron homeostasis during pregnancy. Nutr. 2014;6(8):3062–3083. 10.3390/nu6083062 PMC4145295 25093277

[23] Micronutrients I of M (US) P on. Dietary Reference Intakes for Vitamin A, Vitamin K, Arsenic, Boron, Chromium, Copper, Iodine, Iron, Manganese, Molybdenum, Nickel, Silicon, Vanadium, and Zinc. Diet Ref Intakes Vitam A, Vitam K, Arsenic, Boron, Chromium, Copper, Iodine, Iron, Manganese, Molybdenum, Nickel, Silicon, Vanadium, Zinc. 2001. [cited 3 Feb 2023].

[24] Reddy MB, Cook JD. Assessment of dietary determinants of nonheme-iron absorption in humans and rats. Am J Clin Nutr. 1991;54(4):723–728. 1654740 10.1093/ajcn/54.4.723

[25] Kaufman RM, Pollack S, Crosby WH. Iron-Deficient diet: effects in rats and humans. Blood. 1966;28(5):726–737. 5922678

[26] Fillebeen C, Gkouvatsos K, Fragoso G, et al. Mice are poor heme absorbers and do not require intestinal Hmox1 for dietary heme iron assimilation. Haematologica. 2015;100(9):e334–e337. 25975840 10.3324/haematol.2015.126870 PMC4800685

[27] Ronovsky M, Berger S, Zambon A, et al. Maternal immune activation transgenerationally modulates maternal care and offspring depression-like behavior. Brain Behav Immun. 2017;63:127–136. 27765645 10.1016/j.bbi.2016.10.016

[28] Reisinger SN, Bilban M, Stojanovic T, et al. Lmo3 deficiency in the mouse is associated with alterations in mood-related behaviors and a depression-biased amygdala transcriptome. Psychoneuroendocrinology. 2020;111:104480. 31707294 10.1016/j.psyneuen.2019.104480

[29] Franks B, Curley JP, Champagne FA. Measuring variations in maternal behavior: relevance for studies of mood and anxiety. Neuromethods. 2011;63:209–224.

[30] Yu J, Liu Q, Wang YQ, et al. Electroacupuncture combined with clomipramine enhances antidepressant effect in rodents. Neurosci Lett. 2007;421(1):5–9. 17548153 10.1016/j.neulet.2007.02.052

[31] Der-Avakian A, Markou A. The neurobiology of anhedonia and other reward-related deficits. Trends Neurosci. 2012;35(1):68–77. 22177980 10.1016/j.tins.2011.11.005 PMC3253139

[32] Kim EJ, Monje FJ, Li L, et al. Alzheimer’s disease risk factor lymphocyte-specific protein tyrosine kinase regulates long-term synaptic strengthening, spatial learning and memory. Cell Mol Life Sci. 2013;70(4):743–759. 23007847 10.1007/s00018-012-1168-1 PMC11113176

[33] Crawley J, Goodwin FK. Preliminary report of a simple animal behavior model for the anxiolytic effects of benzodiazepines. Pharmacol Biochem Behav. 1980;13(2):167–170. 10.1016/0091-3057(80)90067-2 6106204

[34] Jones BJ, Roberts DJ. The quantiative measurement of motor inco-ordination in naive mice using an acelerating rotarod. J Pharm Pharmacol. 1968;20(4):302–304. 4384609 10.1111/j.2042-7158.1968.tb09743.x

[35] Porsolt RD, Anton G, Blavet N, et al. Behavioural despair in rats: a new model sensitive to antidepressant treatments. Eur J Pharmacol. 1978;47(4):379–391. 204499 10.1016/0014-2999(78)90118-8

[36] Molendijk ML, de Kloet ER. Coping with the forced swim stressor: current state-of-the-art. Behav Brain Res. 2019;364:1–10. 30738104 10.1016/j.bbr.2019.02.005

[37] Berger S, Ronovsky M, Horvath O, et al. Impact of maternal immune activation on maternal care behavior, offspring emotionality and intergenerational transmission in C3H/He mice. Brain Behav Immun. 2018;70:131–140. 29481858 10.1016/j.bbi.2018.02.008

[38] Pollak DD, Monje FJ, Zuckerman L, et al. An animal model of a behavioral intervention for depression. Neuron. 2008;60(1):149–161. 18940595 10.1016/j.neuron.2008.07.041 PMC3417703

[39] Chen EY, Tan CM, Kou Y, et al. Enrichr: interactive and collaborative HTML5 gene list enrichment analysis tool. BMC Bioinf. 2013;14:1–14. 10.1186/1471-2105-14-128 PMC3637064 23586463

[40] Kuleshov MV, Jones MR, Rouillard AD, et al. Enrichr: a comprehensive gene set enrichment analysis web server 2016 update. Nucleic Acids Res. 2016;44(W1):W90–W97. 27141961 10.1093/nar/gkw377 PMC4987924

[41] Pollak DD, Rey CE, Monje FJ. Rodent models in depression research: classical strategies and new directions. Ann Med. 2010;42(4):252–264. 20367120 10.3109/07853891003769957

[42] Anacker C, Hen R. Adult hippocampal neurogenesis and cognitive flexibility—linking memory and mood. Nat Rev Neurosci. 2017;18(6):335–346. 28469276 10.1038/nrn.2017.45 PMC6261347

[43] Boldrini M, Underwood MD, Hen R, et al. Antidepressants increase neural progenitor cells in the human hippocampus. Neuropsychopharmacology. 2009;34(11):2376–2389. 19606083 10.1038/npp.2009.75 PMC2743790

[44] Berger T, Lee H, Young AH, et al. Adult hippocampal neurogenesis in major depressive disorder and alzheimer’s disease. Trends Mol Med. 2020;26(9):803–818. 32418723 10.1016/j.molmed.2020.03.010

[45] Goodall EF, Heath PR, Bandmann O, et al. Neuronal dark matter: the emerging role of microRNAs in neurodegeneration. Front Cell Neurosci. 2013;7:178. 24133413 10.3389/fncel.2013.00178 PMC3794211

[46] Allen L, Dwivedi Y. MicroRNA mediators of early life stress vulnerability to depression and suicidal behavior. Mol Psychiatry. 2020;25(2):308–320. 31740756 10.1038/s41380-019-0597-8 PMC6974433

[47] Dwivedi Y. Emerging role of microRNAs in major depressive disorder: diagnosis and therapeutic implications. Dialogues Clin Neurosci. 2014;16(1):43–61. 24733970 10.31887/DCNS.2014.16.1/ydwivedi PMC3984890

[48] Li Y, Lin L, Li Z, et al. Iron homeostasis regulates the activity of the microRNA pathway through poly(C)-binding protein 2. Cell Metab. 2012;15(6):895–904. 22633452 10.1016/j.cmet.2012.04.021 PMC3613991

[49] Maccari S, Krugers HJ, Morley-Fletcher S, et al. The consequences of early-life adversity: neurobiological, behavioural and epigenetic adaptations. J Neuroendocrinol. 2014;26(10):707–723. Blackwell Publishing Ltd 25039443 10.1111/jne.12175

[50] Field T. Postpartum depression effects on early interactions, parenting, and safety practices: a review. Infant Behav Dev. 2010;33(1):1–6. 19962196 10.1016/j.infbeh.2009.10.005 PMC2819576

[51] Parent CI, Meaney MJ. The influence of natural variations in maternal care on play fighting in the rat. Dev Psychobiol. 2008;50(8):767–776. 18846499 10.1002/dev.20342

[52] Hellstrom IC, Dhir SK, Diorio JC, et al. Maternal licking regulates hippocampal glucocorticoid receptor transcription through a thyroid hormone–serotonin–NGFI-A signalling Cascade. Philos Trans R Soc B Biol Sci. 2012;367:2495. 10.1098/rstb.2012.0223 PMC3405683 22826348

[53] Febo M, Stolberg TL, Numan M, et al. Nursing stimulation is more than tactile sensation: it is a multisensory experience. Horm Behav. 2008;54(2):330–339. 18440003 10.1016/j.yhbeh.2008.02.024 PMC4915061

[54] Liu D, Diorio J, Tannenbaum B, et al. Maternal care, hippocampal glucocorticoid receptors, and hypothalamic- pituitary-adrenal responses to stress. Science. 1997;277(5332):1659–1662. 9287218 10.1126/science.277.5332.1659

[55] Rilling JK, Young LJ. The biology of mammalian parenting and its effect on offspring social development. Science American association for the advancement of. Science. 2014;345(6198):771 6. 25124431 10.1126/science.1252723 PMC4306567

[56] Albacar G, Sans T, Martín-Santos R, et al. An association between plasma ferritin concentrations measured 48 h after delivery and postpartum depression. J Affect Disord. 2011;131(1-3):136–142. 21130499 10.1016/j.jad.2010.11.006

[57] Beard JL, Hendricks MK, Perez EM, et al. Maternal iron deficiency anemia affects postpartum emotions and cognition. J Nutr. 2005;135(2):267–272. 15671224 10.1093/jn/135.2.267

[58] Alharbi AA, Abdulghani HM. Risk factors associated with postpartum depression in the saudi population. Neuropsychiatr Dis Treat. 2014;10:311–316. 24570584 10.2147/NDT.S57556 PMC3933724

[59] Milman N. Anemia–still a major health problem in many parts of the world. !Ann Hematol. 2011;90(4):369–377. 21221586 10.1007/s00277-010-1144-5

[60] Etebary S, Nikseresht S, Reza Sadeghipour H, et al. Postpartum depression and role of serum trace elements. Iran J Psychiatry. 2010;5:40. 22952489 PMC3430492

[61] Bin KI, Park SC. Neural circuitry–neurogenesis coupling model of depression. Int J Mol Sci. 2021;22:1–18. 10.3390/ijms22052468 PMC7957816 33671109

[62] Sahay A, Hen R. Adult hippocampal neurogenesis in depression. Nat Neurosci. 2007;10:1110–1115. [cited 2 Feb 2023]. 10.1038/nn1969 17726477

[63] Nieto-Quero A, Chaves-Peña P, Santín LJ, et al. Do changes in microglial status underlie neurogenesis impairments and depressive-like behaviours induced by psychological stress? A systematic review in animal models. Neurobiol Stress. 2021;15:100356. 34355047 10.1016/j.ynstr.2021.100356 PMC8319800

[64] Petri R, Malmevik J, Fasching L, et al. miRNAs in brain development. Exp Cell Res. 2014;321(1):84–89. 24099990 10.1016/j.yexcr.2013.09.022

[65] Issler O, Chen A. Determining the role of microRNAs in psychiatric disorders. Nat Rev Neurosci. 2015;16(4):201–212. 25790865 10.1038/nrn3879

[66] Zurawek D, Kusmider M, Faron-Gorecka A, et al. Time-dependent miR-16 serum fluctuations together with reciprocal changes in the expression level of miR-16 in mesocortical circuit contribute to stress resilient phenotype in chronic mild stress - An animal model of depression. Eur Neuropsychopharmacol. 2016;26(1):23–36. 26628105 10.1016/j.euroneuro.2015.11.013

[67] Fonken LK, Gaudet AD, Gaier KR, et al. MicroRNA-155 deletion reduces anxiety- and depressive-like behaviors in mice. Psychoneuroendocrinology. 2016;63:362–369. 26555429 10.1016/j.psyneuen.2015.10.019 PMC13014412

[68] Li Y, Li S, Yan J, et al. miR-182 (microRNA-182) suppression in the hippocampus evokes antidepressant-like effects in rats. Prog Neuropsychopharmacol Biol Psychiatry. 2016;65:96–103. 26368940 10.1016/j.pnpbp.2015.09.004

[69] Ronovsky M, Zambon A, Cicvaric A, et al. A role for miR-132 in learned safety. Sci Rep. 2019;9(1):528. 10.1038/s41598-018-37054-z PMC6346013 30679653

[70] Watkeys OJ, Kremerskothen K, Quidé Y, et al. Glucocorticoid receptor gene (NR3C1) DNA methylation in association with trauma, psychopathology, transcript expression, or genotypic variation: a systematic review. Neurosci Biobehav Rev. 2018;95:85–122. 30176278 10.1016/j.neubiorev.2018.08.017

[71] Schmidt M, Lax E, Zhou R, et al. Fetal glucocorticoid receptor (Nr3c1) deficiency alters the landscape of DNA methylation of murine placenta in a sex-dependent manner and is associated to anxiety-like behavior in adulthood. Transl Psychiatry. 2019;9:23. 30655507 10.1038/s41398-018-0348-7 PMC6336883

[72] Lin Z, Su Y, Zhang C, et al. The interaction of BDNF and NTRK2 gene increases the susceptibility of paranoid schizophrenia. PLoS One. 2013;8(9):e74264. 24069289 10.1371/journal.pone.0074264 PMC3775790

[73] Adams JH, Wigg KG, King N, et al. Association study of neurotrophic tyrosine kinase receptor type 2 (NTRK2) and childhood-onset mood disorders. Am J Med Genet B Neuropsychiatr Genet. 2005;132B(1):90–95. 15389758 10.1002/ajmg.b.30084

[74] Tao R, Li C, Jaffe AE, et al. Cannabinoid receptor CNR1 expression and DNA methylation in human prefrontal cortex, hippocampus and caudate in brain development and schizophrenia. Transl Psychiatry. 2020;10(1):158. 32433545 10.1038/s41398-020-0832-8 PMC7237456

[75] Navarro D, Gasparyan A, Navarrete F, et al. Molecular alterations of the endocannabinoid system in psychiatric ­disorders. Int J Mol Sci 2022, Vol 23, Page 4764. 2022;23:4764. 35563156 10.3390/ijms23094764 PMC9104141

