# Supplementary figures and images for: Early-life iron deficiency persistently disrupts affective behaviour in mice
Source: Ann Med. 2023 Apr 25;55(1):1265–77. doi: 10.1080/07853890.2023.2191003 (PMC10132221; doi:10.1080/07853890.2023.2191003)

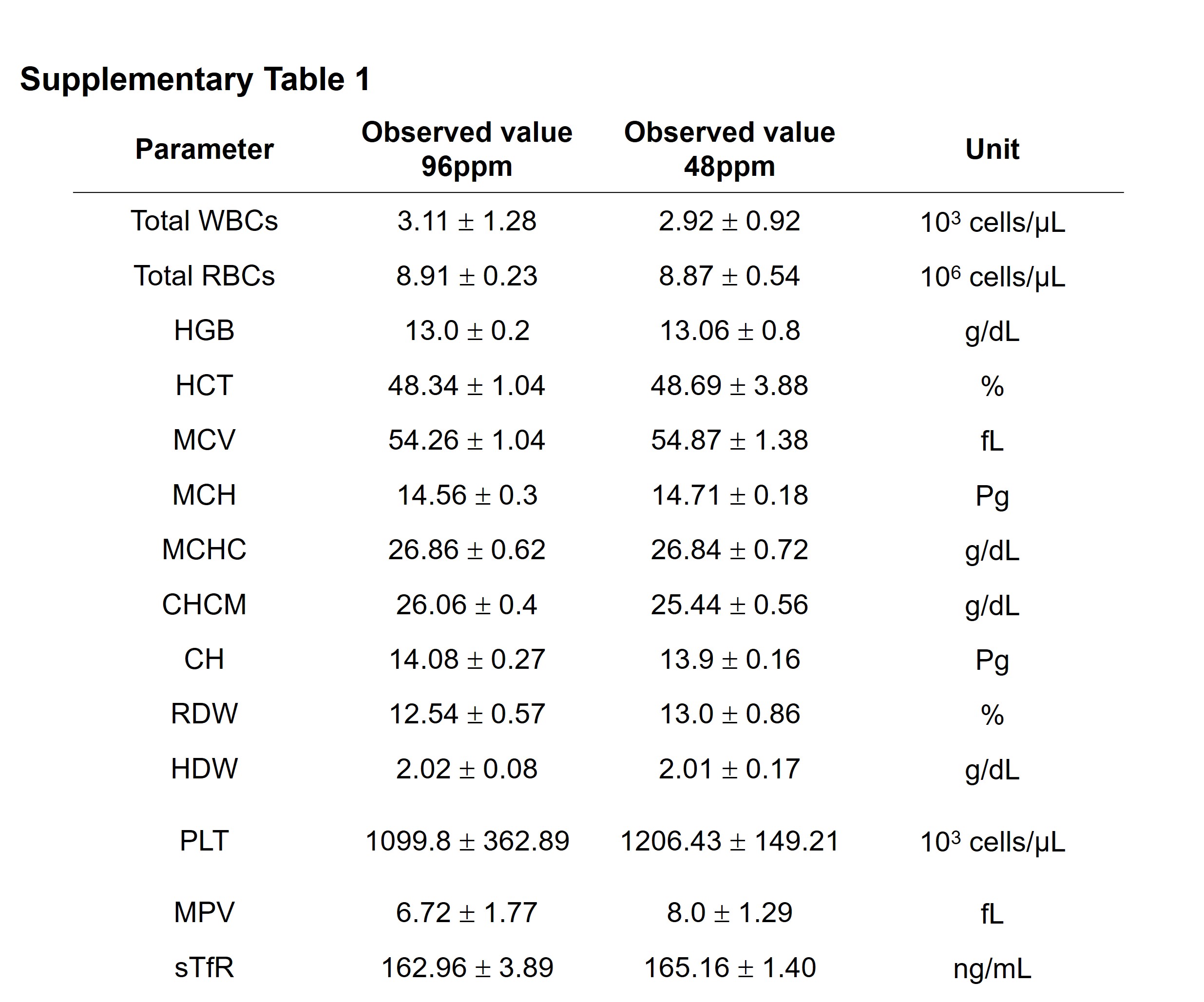

Supplement: Supplemental Material [file IANN_A_2191003_SM9143.jpg]
